# Homogeneous and Reproducible Mixing of Highly Viscous Biomaterial Inks and Cell Suspensions to Create Bioinks

**DOI:** 10.3390/gels7040227

**Published:** 2021-11-23

**Authors:** Finn Dani, Tilman Ahlfeld, Franziska Albrecht, Sarah Duin, Petra Kluger, Anja Lode, Michael Gelinsky

**Affiliations:** 1 Centre for Translational Bone, Joint and Soft Tissue Research, Faculty of Medicine, Technische Universität Dresden, 01307 Dresden, Germany; finn.dani@tu-dresden.de (F.D.); tilman.ahlfeld@tu-dresden.de (T.A.); sarah.duin@tu-dresden.de (S.D.); anja.lode@tu-dresden.de (A.L.); 2 Reutlingen Research Institute, Reutlingen University, 72762 Reutlingen, Germany; Franziska.Albrecht@Reutlingen-University.de (F.A.); petra.kluger@reutlingen-university.de (P.K.)

**Keywords:** bioprinting, plotting, highly viscous bioink, methylcellulose, plasma, gellan gum, static mixer

## Abstract

Highly viscous bioinks offer great advantages for the three-dimensional fabrication of cell-laden constructs by microextrusion printing. However, no standardised method of mixing a high viscosity biomaterial ink and a cell suspension has been established so far, leading to non-reproducible printing results. A novel method for the homogeneous and reproducible mixing of the two components using a mixing unit connecting two syringes is developed and investigated. Several static mixing units, based on established mixing designs, were adapted and their functionality was determined by analysing specific features of the resulting bioink. As a model system, we selected a highly viscous ink consisting of fresh frozen human blood plasma, alginate, and methylcellulose, and a cell suspension containing immortalized human mesenchymal stem cells. This bioink is crosslinked after fabrication. A pre-crosslinked gellan gum-based bioink providing a different extrusion behaviour was introduced to validate the conclusions drawn from the model system. For characterisation, bioink from different zones within the mixing device was analysed by measurement of its viscosity, shape fidelity after printing and visual homogeneity. When taking all three parameters into account, a comprehensive and reliable comparison of the mixing quality was possible. In comparison to the established method of manual mixing inside a beaker using a spatula, a significantly higher proportion of viable cells was detected directly after mixing and plotting for both bioinks when the mixing unit was used. A screw-like mixing unit, termed “HighVisc”, was found to result in a homogenous bioink after a low number of mixing cycles while achieving high cell viability rates.

## 1. Introduction

Extrusion-based bioprinting is characterized by the continuous release of bioinks through a printing nozzle. By robotic execution, a three-dimensional (3D) construct can be fabricated by ordered arrangement of the extruded bioink strands; this process is called 3D (bio-)plotting [1]. The bioink consists of two components: the biomaterial ink and the cells. Over time, two main categories of bioinks became important.

The first category are bioinks with low viscosity. These bioinks need a high technological effort for processing, otherwise they would collapse after extrusion [2]. For example, in situ crosslinking techniques such as nebulisation of calcium chloride solution [3] in the case of alginate and other ionically cross-linkable bioinks or UV exposure in the case of inks modified with photoreactive chemical groups were successfully applied [4]. Other bioinks of low viscosity take usage of support baths, which are sacrificed during post-processing [5,6]. These technical efforts are limiting the number of processable materials while solutions for challenges such as layer delamination and post-processing of the plotted bioink constructs are still under development. Another application of low viscosity bioinks is their combination with mechanically supporting materials such as poly-ε-caprolactone [7,8] or calcium phosphate cements [9,10], which is often conducted in bone and cartilage tissue engineering research.

The second category are bioinks with high viscosity, which allow the direct fabrication of volumetric constructs in air. The stabilisation of such plotted constructs is implemented after the extrusion process since the bioink is capable of withstanding collapses caused by gravity. Commonly, such bioinks lack a good biological performance since their viscosity is increased by an impractical enhancement of their solid content. However, the rheological properties, such as viscosity and shear recovery can be improved and tailored to specific needs by modulation of the polymer network [11] or by combination with reactive filler materials [12]. High viscosity bioinks can easily be processed in combination with other bioinks or biomaterial inks by multichannel plotting and they enable the fabrication of constructs with defined macropores, which might be beneficial for nutrient and oxygen supply in a volumetric biofabricated construct. In the last few years, many groups developed bioinks of the second category which show good biological response despite their high viscosity (in non-flowing state), mainly for bone and cartilage regeneration, but also in soft tissue applications or as wound filler for deep skin burns [13,14,15]. Highly viscous bioinks possess a potentially high polymer content, preventing the crucial problem of sedimentation of cells or particles due to a closer polymer net compared to low viscous bioinks which are impaired by cell sedimentation [16]. While for low viscosity bioinks, the timeframe of cell sedimentation lies within a range of a few minutes, being relevant for bioprinting; these times are significantly higher for highly viscous bioinks [16]. While there are demanding technical solutions addressing the sedimentation in low viscosity bioinks, highly viscous bioinks offer a clear advantage in this regard [17].

One example of the second category is a blend of alginate (alg) and methylcellulose (mc) [18,19]. Here, the methylcellulose enhances the viscosity of the biomaterial ink to achieve the accurate deposition of strands. The performance of this blend could be enhanced by the addition of human blood plasma [10]. The plasma-alg-mc bioink combines the good shape fidelity of alg–mc blends with the favourable biological properties of blood plasma, which is a pool of several proteins, especially fibrinogen [10,20] and growth factors. This ink showed favourable properties in shear recovery and shear thinning tests.

Specific bioinks made from a gellan gum solution can be attributed to the second category as its thermo-reversible gel-forming behaviour leads to the possibility of using it as a high viscosity ink when handled at room temperature [21]. Furthermore, the material can be gelled in the presence of millimolar concentrations of cations, e.g., calcium and magnesium ions in cell culture medium, leading to a stable hydrogel independent of thermal conditions [22,23]. Through the combination of these two gelling mechanisms, a shape fidelity comparable to those of alg–mc gels can be achieved.

Although bioinks of both categories are of great interest for the biofabrication field, one essential part of their preparation was only optimised for low viscosity bioinks: the mixing of cells and the (bio)polymer sol forming the bioink. Commercially available products take usage of a static mixer which allows uniform and homogeneous mixing of the cell suspension (due to a rheological behaviour similar to that of water) and the low viscosity bioink. This is presumably caused by a turbulent flow of the two components within the static mixer. Bioinks of higher viscosity do not reach the level of turbulent flow but stay in laminar flow during the mixing and thus cannot be processed by these tools. Due to this reason, cells are usually mixed manually in open containers with the biomaterial ink using a spatula or a pipette, which causes problems in reproducibility, creates air bubbles, and it influences the viability of cells. This might be problematic when such bioinks need to be prepared under good manufacturing practice (GMP) conditions or in environments with high-security regulations such as the International Space Station (ISS) [24,25].

Herein, we investigated whether specific static mixers operating under a laminar flow regime are suitable for mixing of cell suspensions and high viscosity inks by using plasma-alg-mc as a model ink. To prove the applicability of the mixer independently of the biomaterial ink, in addition a gellan-based ink that possesses different rheological and chemical properties was used. The mixing units can be fabricated cost-effectively using a standard stereolithography (SLA) printer and have two Luer lock adapters to connect them to two standard syringes, making the system easily applicable in any laboratory. We investigated homogeneity, cell viability and reproducibility of the mixed bioinks and figured out an optimal variant. This paper should enable many researchers to create homogenously mixed high viscosity bioinks for their experiments.

## 2. Results

### 2.1. Mixing Procedure and Mixer Designs

**Figure 1:**
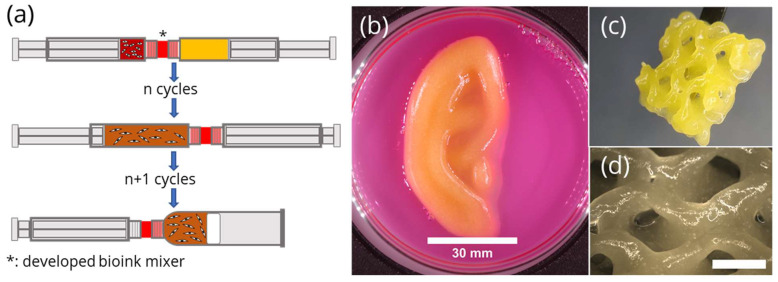
(**a**) Schematic of the mixing process: cell suspension (left) and biomaterial ink (right) in separate syringes before mixing, after n mixing cycles and after n+1 mixing cycles (to transfer the bioink into a plotting cartridge); (**b**) 3D-plotted ear-like structure made from the plasma-alg-mc bioink model, scale bar 30 mm; (**c,d**) gyroid structure made from plasma-alg-mc bioink model, (**d**) scale bar: 5 mm.

An easy and reproducible way of mixing cell suspension and biomaterial ink already exists for low viscosity bioinks [26] by having both liquids in separate syringes, merging them by the use of a conventional static mixer comprising several serial helical mixing units. For bioinks of high viscosity, a similar strategy was investigated here where two syringes containing either cell suspension or a plasma-alg-mc-ink are connected by an additively manufactured static mixer between them (Figure 1a). To reduce the dead volume between both syringes, only one mixer element was included, thus relying on several mixing cycles between both syringes to ensure homogenous mixing results. Examples for fabricated constructs using plasma-alg-mc and cell culture medium as a high-viscosity bioink model are shown in Figure 1b–d. The ear (Figure 1b) and the gyroid structure (Figure 1c,d) were plotted in air without any supporting structure and no collapses were observed. This is expected for high, but not for low, viscous bioinks.

**Figure 2:**
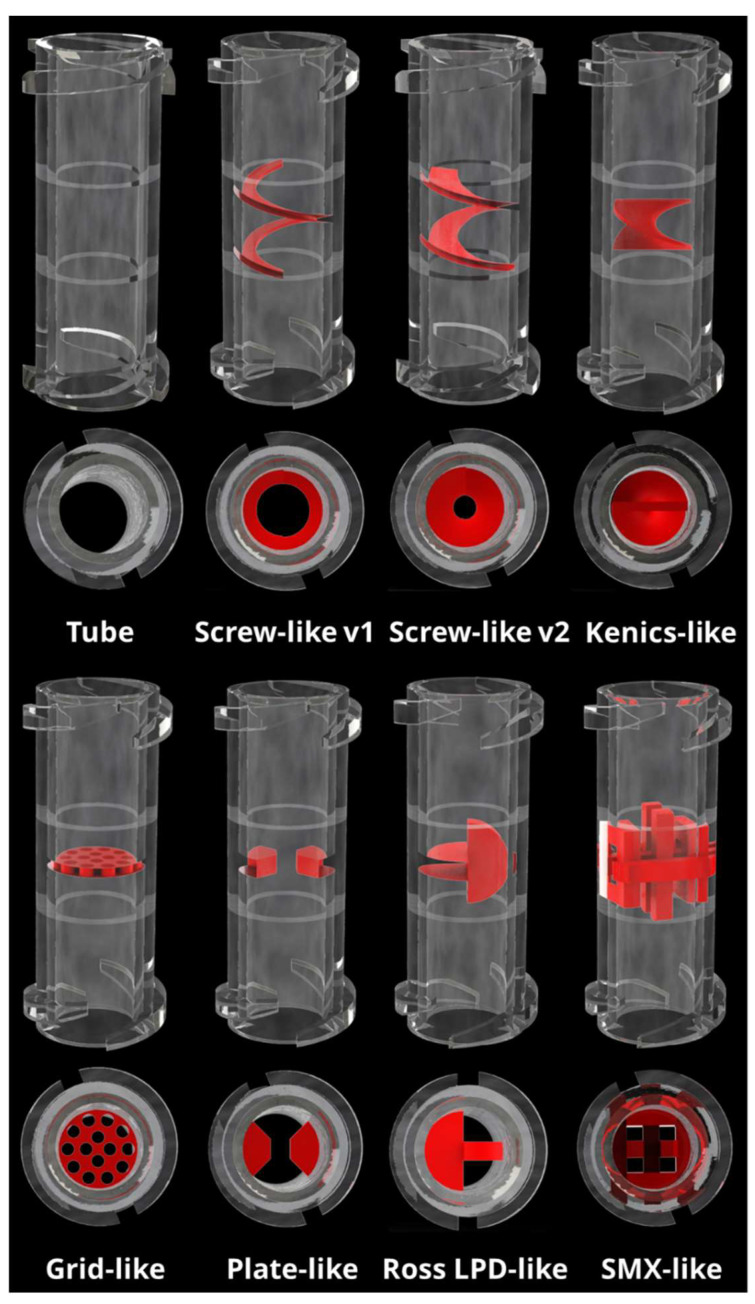
3D models of all designs used for static mixing units in this work. The Luer lock on both ends ensures compatibility with standard syringes and plotter cartridges.

The connectors consisting of the mixing unit and a Luer lock system on each side were fabricated using a stereolithography printer to achieve a part that features a high resolution and is resistant to high temperatures and pressures allowing sterilization by an autoclaving process. Several mixer designs were selected for testing after evaluating commonly used methods for the static mixing of highly viscous fluids with low viscosity fluids. The designs followed either two- or three-dimensional characteristics and were selected and partially modified to fit the spatial parameters (Figure 2).

The first design (“Tube”) consists of just the connector without a mixer element with an inner diameter of 4.3 mm and a length of 18.62 mm, including the Luer locks. This geometry leads to an inner volume of 0.2704 mL. The second set of designs took usage of helical structures. The resulting structures “Screw-like v1” and “Screw-like v2” are based on the same helix with a lead of 3.33 mm and 1.5 rotations. They differ in the depth of the mixer inside the connector; while the structure of “v1” only has a depth of 0.7 mm, for “v2” it was increased to 1.5 mm leading to an inner aperture of 2.9 mm and 1.3 mm, respectively. As for the third helical structure, a “Kenics-like” helix was fabricated, reiterating a usual Kenics static mixer [27]; it has a lead of 5.06 mm at 0.5 rotations. The third set of designs investigated two-dimensional apertures: “Grid-like”, featuring 14 holes, each with a diameter of 0.7 mm and “Plate-like” which leaves an opening of 1.74 mm between two blades. For a fourth set, two different 3D-designs were developed. “Ross LPD-like” and “SMX-like” static mixers were inspired by respective existing static mixers [28] that were already described in literature.

### 2.2. Investigations on the Homogeneity during the Mixing Process

**Figure 3:**
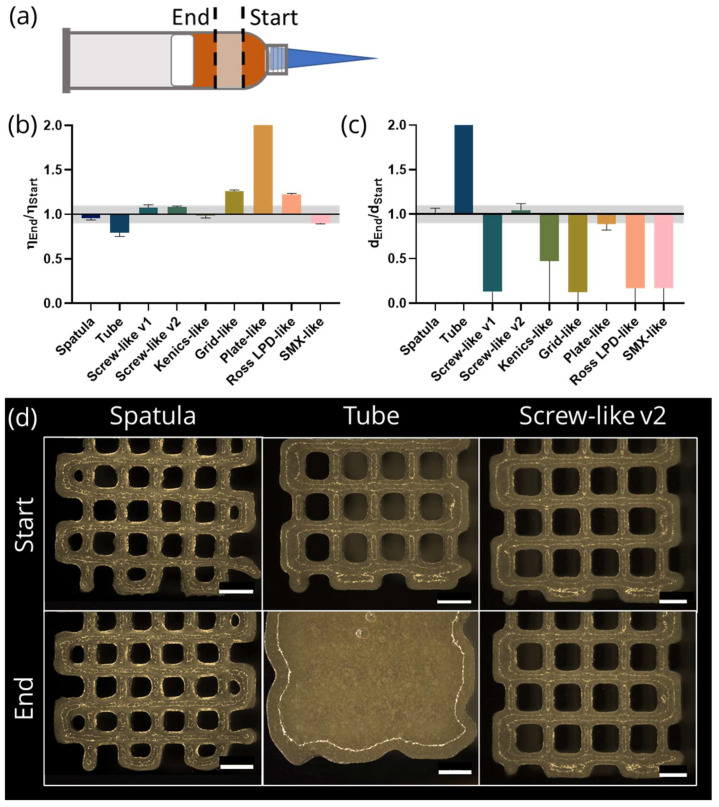
(**a**) Schematic of printing needle to show the sections used for analysis; (**b**) homogeneity as calculated by the ratio of viscosities η, n_Start_, n_End_ = 3, mean ± standard error; (**c**) shape fidelity determined by the ratio of strand widths d of plotted scaffolds, n_Start_, n_End_ = 60, mean ± standard deviation; (**d**) plotted four-layered scaffolds fabricated with the plasma-alg-mc bioink model from start and end of cartridge. Scale bars: 2 mm.

**Figure 4:**
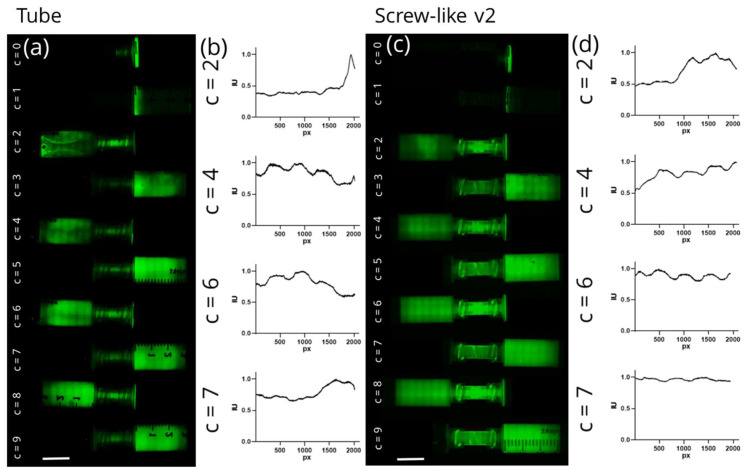
Mixing of plasma-alg-mc biomaterial ink with green fluorescent protein dissolved in cell culture medium using “Tube” and “Screw-like v2” mixer unit; (**a**,**c**): fluorescence microscopic images of both syringes before mixing (c = 0) and after several mixing steps (c = 1–9). (**b**,**d**): normalized intensity graphs for “Tube” and “Screw-like v2” mixing unit as measured over the entire cartridge length. Scale bars: 15 mm.

In preliminary experiments (results not shown), six mixing cycles were specified as the minimal amount necessary for homogenous results by visual observation. For the following systematic assessment of homogeneity, three different methods were chosen: the ratio of viscosities and strand widths, respectively (shown in Figure 3), as well as an optical measurement using green fluorescent protein (GFP, depicted in Figure 4). To determine ratios of viscosity and shape fidelity, a first volume of material was taken from the syringe/plotting cartridge (“Start”) after the aforementioned six mixing steps and was either plotted to measure the width of a strand or analysed in a rotational rheometer. Then, as shown in Figure 3a, one third of the cartridge’s ink content was discarded and with the remaining ink (“End”), the same investigations were conducted.

The rheological measurements were carried out as described in Chapter 5.5.1. After obtaining the viscosity data for both the start and the end section of the biomaterial ink, the ratio of η_End_/η_Start_ was determined; a value of 1 thus corresponds to the optimum mixing quality. Therefore, a ratio greater than 1 indicates that the material at the start of the syringe has a lower viscosity than the material at the end. Accordingly, a ratio lower than 1 describes the opposite effect. A deviation of ±10% was defined as an acceptable divergence from the ideal (Figure 3b, grey area). The generally applied mixing method using a spatula was performed as a control group. It is noteworthy that all three mixer units using helical structures (“Screw-like” v1 and v2 and “Kenics-like”) result in viscosity ratios close to 1, demonstrating very good mixing quality. Both two-dimensional mixers, “Plate-like” and “Grid-like”, on the other hand, result in high ratios, the mixing units inspired by Ross-LPD and SMX also do not lead to acceptable mixing quality. The spatula-mixed control group showed uniform viscosities as expected.

Figure 3c shows the graph obtained from measuring the widths of plotted strands. In the same procedure as for the rheological data, the strand widths (d) of scaffolds plotted with material from either the start or the end of a plotting cartridge were measured and the ratio of d_End_/d_Start_ was calculated. Accordingly, the ideal ratio was achieved at a value of 1. A ratio above 1 indicated wider strands in scaffolds plotted with material from the end of the cartridge, while a ratio below 1 was caused by these strands being narrower than their counterpart. Using the spatula for manual mixing leads to uniform scaffolds with no differences between start and end. In contrast, a clear lack in homogeneity is noticeable between the material at the start and end when mixed using a “Tube” unit. Despite having achieved viscosity ratios close to 1, material from several mixer units (“Screw-like”, “Kenics-like” and “SMX-like”) produced considerably wider strands plotted using material from the start than at the end. It can be concluded that the mixing efficiency in this mixing units is unsatisfactory, leading to inhomogeneous biomaterial inks. Only the “Screw-like v2” and “Plate-like” mixing units were able to obtain results within the defined interval of ±10 %. Taking both measurements into account, the “Screw-like v2” mixing unit was selected as a favourite for further experiments. The difference in strand sizes when using the “Tube” unit vs. the “Screw-like” unit or a spatula is very obvious when observing the scaffolds (Figure 3d).

To visualize the mixing efficiency after various mixing steps, the worst (“Tube”) and best (“Screw-like v2”) units were chosen for an experiment to monitor the development of homogeneity using GFP as fluorescent dye within the cell culture medium. For both mixing units, the biomaterial ink was imaged before the first and consequently after each new mixing step (see Figure 4a,c).

Both mixing units show similar behaviour before and after the first mixing cycle (number of cycles will be defined by the variable n). At n = 0, only the fluorescent dye in one syringe is visible. At n = 1, the biomaterial ink was pressed into the same syringe without mixing. Only a slight smearing of the dye on the walls of the syringe is visible. At n = 2, a first mixing of both liquids is noticeable; however, images and intensity graphs (Figure 4b,d) for both mixing units show an inhomogeneous distribution of fluorescent dye. The “Tube” unit then shows some improvement in homogeneity until mixing step 9. However, in the intensity graph, different intensities are still noticeable indicating that homogeneity was not achieved by mixing step 7. In contrast, homogenous mixing after six mixing cycles was proven for the “Screw-like v2” mixing unit. The clear differences in intensity between even and odd numbers of mixing cycles can be attributed to the initial presence of fluorescent dye in the right syringe. Over the first five mixing cycles, before homogeneity was achieved, the dye either flows along the outer walls inside the right syringe or was trapped inside the dead volume of the syringe when the biomaterial ink was pressed into the left syringe. The local minima/maxima visible throughout all fluorescent images and intensity plots are an artefact of the stitching mode of the microscope and should not be overestimated. With these measurements, the early observation that homogeneity was achieved after six mixing cycles could be further evidenced.

### 2.3. Cell Survival

**Figure 5:**
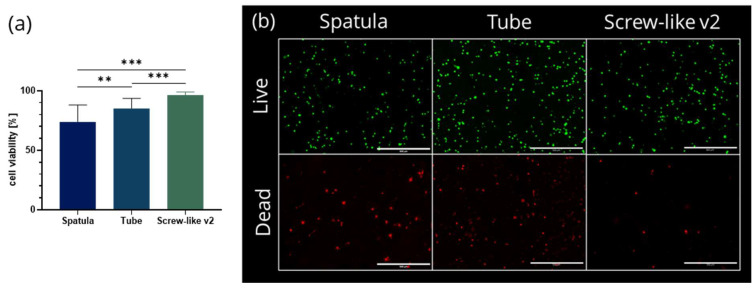
(**a**) Ratio of viable cells and total cell number (n ≥ 9, mean ± standard deviation, ** *p* < 0.01, *** *p* < 0.001) and (**b**) representative images showing living and dead cells for the mixing with spatula, “Tube” unit and “Screw-like v2” mixer unit taken directly after printing and crosslinking of plasma-alg-mc scaffolds. Green = metabolically active cells, red = dead cells, scale bar: 500 µm.

After selecting the optimal mixer for homogeneity, cell survival during the mixing process was investigated by plotting cell-laden constructs using the same scaffold design as described in Chapter 2.2. The viability results for the “Screw-like v2” mixer unit were compared to those of the “Tube” unit and the spatula-mixed control, taking fluorescence images of the same three positions on each scaffold, one within a strand crossing and two on separate strands. For both mixing units, a significantly higher and more reproducible initial cell viability after mixing and plotting was shown than compared to the cells in the bioink mixed with the spatula method. In a direct comparison of both mixing units, a significantly higher cell viability was found when mixing with the “Screw-like v2” mixer, resulting in 85.3 ± 8.3% viable cells using the “Tube” unit and 96.4 ± 2.6% using the “Screw-like v2” mixer (Figure 5). In contrast, the spatula method resulted in cell viabilities of 74.1 ± 14.0%. The cell viabilities using other mixers laid in between 70–90% (Figure S1).

### 2.4. Validation of the Static Mixer Using a Highly Viscous Gellan-Based Bioink

A gellan gum-based bioink was used to demonstrate the applicability of the chosen mixing unit for a wide range of highly viscous biomaterial inks. Therefore, all experiments concerning rheology, shape fidelity and cell survival were repeated using this second biomaterial ink and the “Screw-like v2” static mixer as previously identified optimal mixing unit. The mixing quality of both bioinks after six mixing steps was then compared to gain insights regarding the dependency of mixing results on the hydrogel type.

Since the Ca^2+^ ion concentration in cell culture medium is high enough to crosslink the gellan gum [22], the crosslinking process began with the first mixing step, gelling the ink during the mixing process. Therefore, the viscosity is rising with each mixing step and with increasing mixing time, resulting in a vastly different fluid behaviour compared to the alginate-based ink.

**Figure 6:**
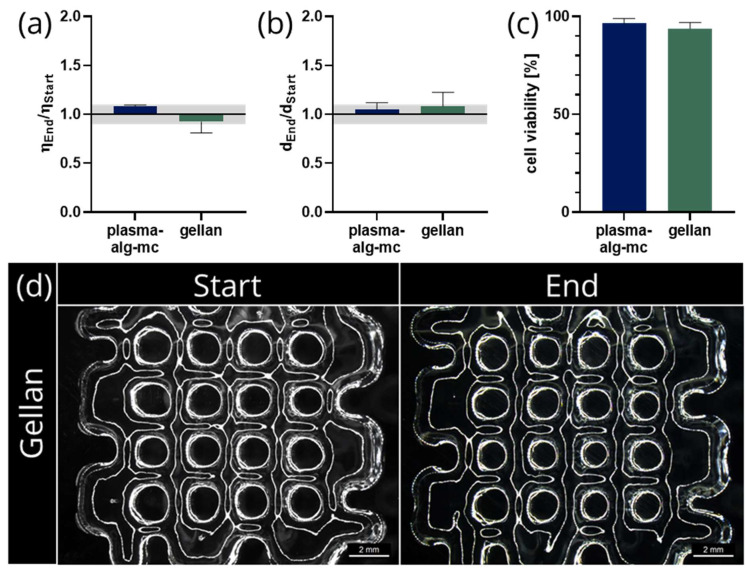
(**a**) Homogeneity as calculated by the ratio of viscosities η, n_Start_, n_End_ = 3, mean ± standard error; (**b**) shape fidelity determined by the ratio of strand widths d of plotted scaffolds, n_Start_, n_End_ = 60, mean ± standard deviation; (**c**) ratio of viable cells and total cell number (n ≥ 9, mean ± standard deviation); (**d**) plotted four-layered scaffolds fabricated with the gellan bioink from start and end of cartridge. Scale bars: 2 mm.

To ensure comparability between both inks, the mixing parameters of six cycles and a mixing time of 20 s per cycle were kept unchanged. Due to the gellan gum-bioink being still in development, there is no standard procedure of mixing that could be used as a reference group. Therefore, all results are compared to the performance of the mixer using the plasma-alg-mc biomaterial ink. The results were analysed according to previous experiments that are described in Chapters 2.2 and 2.3. Regarding the ratio of viscosities of the biomaterial ink from different sections of the cartridge, shown in Figure 6a, the results for the gellan-based bioink are within the defined area of divergence and similar to the ratio achieved by the plasma-alg-mc ink, indicating a good mixing quality. This finding is further evidenced by the ratio of strand widths, shown in Figure 6b, as well as the microscopy image of plotted constructs (Figure 6d). When investigating the viability of MSC after the mixing process and plotting, no significant difference between the cells embedded in plasma-alg-mc or gellan-based bioink was evident (Figure 6c), demonstrating that even the crosslinking starting during the mixing process was not detrimental to cell survival.

**Figure 7:**
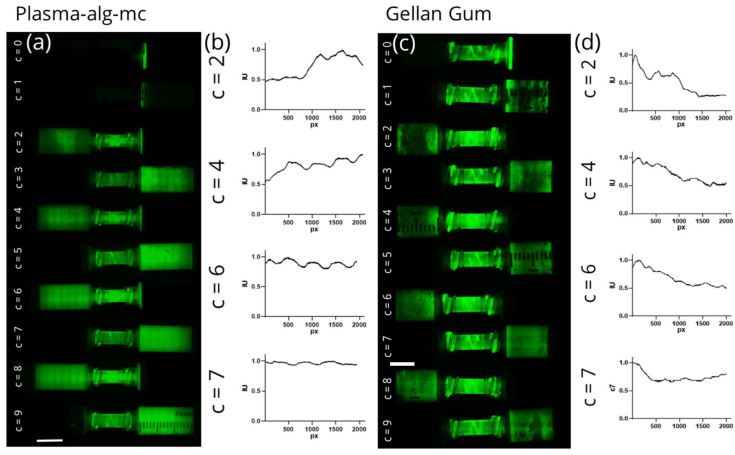
Mixing of plasma-alg-mc and gellan-based biomaterial ink with green fluorescent protein dissolved in cell culture medium using the “Screw-like v2” mixer unit; (**a**,**c**): fluorescence microscopic images of both syringes before mixing (c = 0) and after several mixing steps (c = 1–9). (**b**,**d**): normalized intensity graphs measured over the entire cartridge length. Scale bar: 15 mm.

Figure 7 demonstrates the mixing quality of the “Screw-like v2” mixer using both introduced biomaterial inks. As described previously, green fluorescent protein was diluted in crosslinking medium and then filled into one syringe. The other syringe was filled with 3% gellan biomaterial ink. Using fluorescence microscopy, the mixing construct was imaged before the first and after each subsequent mixing step. In comparison with the plasma-alg-mc ink, the pre-crosslinking of gellan gum directly after contact with the crosslinking medium leads to a different mixing behaviour and a slightly delayed homogenization. After six mixing steps, there is still a slight gradient over the length of the syringe; however, homogeneity is achieved after seven mixing cycles.

## 3. Discussion

While low viscosity bioinks need complex technical solutions to maintain shape fidelity which limits the choice of materials for bioprinting, one of their advantages lies in the established mixing protocols and available mixing devices [2]. Highly viscous bioinks on the other hand overcome these material difficulties and are plottable in air without any support. However, no standardized mixing system has been developed for these bioinks so far, which may lead to non-reproducible results. Herein, we developed a novel approach for reproducible and homogenous mixing of highly viscous bioinks using an additively manufactured mixing unit. Several different versions of mixing units were investigated, and “Screw-like v2” was finally selected as the mixing unit showing the best properties regarding viscosity, shape fidelity, homogeneity, and cell survival. These four parameters were selected to create a holistic picture of the functionality of the mixer and the resulting bioink. Since it is evidenced that a cell density in the applicable order of magnitude has no impact on the rheological behaviour [29,30,31] and plottability of bioinks, experiments regarding the material properties of the bioink were carried out using a cell-free biomaterial ink mixed with cell culture medium acting as a bioink model.

Generally, the best mixing results are achieved when a turbulent flow regime is prevalent, and thus static mixers for low viscous bioinks are built under these principles [26]. For highly viscous Newtonian liquids it is known, however, that the mixing process needs to be effective under a laminar flow regime [32,33], relying on increasing the interface surface between both liquids. Even though both bioinks used herein are highly viscous non-Newtonian liquids, a similar mixing behaviour was assumed while designing the mixing units [31]. To reduce the dead volume caused by the mixer, we decided against several mixer units within one mixer and instead implemented several mixing cycles using only one compact mixer unit.

Mixing units for low viscous bioinks usually contain a series of helical mixers, resulting in both a good homogeneity and high cell survival during mixing [26]. In accordance, under the mixer designs investigated here, there were several helically shaped units which demonstrated promising results for the mixing of high viscosity bioinks. The number of mixing cycles had to be investigated under two opposite aspects: (i) the mixing quality improves with a higher number of mixing cycles, (ii) a higher number in mixing cycles leads to the increased duration of shear stress on the suspended cells. Even though better mixing results were obtained when applying more mixing steps, improving the optical homogeneity up to the ninth mixing cycle, six cycles were deemed to provide the best results regarding cell survival.

In summary, it was ascertained that the homogeneity of a bioink can be described by the ratio of the rheological properties and the ratio of the plottability of the bioink in different zones of the mixing unit. By investigating the viscosity and shape fidelity in both defined zones (“Start” and “End”), it was found that the shape fidelity was a more sensitive indicator of inhomogeneous mixing quality than the viscosity alone. The obtained results were confirmed by analysing the visual homogeneity through monitoring the distribution of green fluorescent protein (GFP) over the mixing cycles (Figure 4). GFP, with a molecular weight of 26.9 kDa, is three orders of magnitude smaller than the average mesenchymal stem cell with a diameter of approximately 30 µm, which could result in a different mixing behaviour of cell suspensions compared to GFP-solution [34,35]. While these differences were not directly investigated, a homogenous cell distribution within the plotted constructs and a good shape fidelity strongly suggest that our mixing model is accurate.

It was concluded that “Screw-like v2” showed the best mixing results in all three experiments and we want to propose the term “HighVisc”-mixer for this design.

Cell viability rates in plasma-alg-mc after mixing were, except for one mixer model, higher than the survival rates achieved using the spatula mixing method. In the literature, there is evidence suggesting a detrimental effect on the viability of MSC when they are subjected to prolonged periods of high shear stress [31,36]. It can be hypothesized that a single cell within the mixing unit experiences high shear stress only for a short span of time and, depending on the number of mixing cycles, only for a limited number of times. Using spatula mixing, which might also take 120 s to perform, each cell is subjected to more irregular, possibly longer uninterrupted times of shear stress. Of course, spatula mixing can be highly dependent on one’s handling and skill and 80–90% cell viability can be achieved using this method [10,37]. However, using a mixing device is not only more convenient, but it can level out varying skill levels and handling procedures of different users.

The bioink chosen for initial assessment of the mixer units was characterized well [10,38]. To evaluate the practicability of the mixing units for a wider range of applications and users, a different bioink was selected to validate the previous findings and possibly identify modifications to the mixing unit and protocol than may be required when applied to different inks. The gellan-based biomaterial ink differs significantly from plasma-alg-mc regarding its crosslinking state and flow properties. At 37 °C, both the gellan solution and the crosslinking medium are water-like liquids with a very low viscosity. At room temperature, however, the gellan solution stabilises, forming a highly viscous gel. This bioink was chosen for several reasons: its high viscosity at room temperature, indicating good plotting properties [39], its different form of crosslinking that already starts during the mixing process and its high cytocompatibility. These properties allow for the comparison of both bioinks in every method of qualifying the mixing process introduced here. Small differences were found in the handling of the mixer for this material. Since there is a noticeable pre-crosslinking initiated by calcium and magnesium ions within the cell culture medium [22], more force was needed to move the material through the mixing unit. Additionally, the period for one mixing cycle cannot be expanded as much as it is possible with the plasma-alg-mc ink due to an increasing viscosity, lowering the mixing efficiency at a low flow velocity. These differences in material properties led to an additional mixing cycle required to obtain a homogenous bioink. It can be assumed that a higher material flow during the mixing (e.g., shorter mixing cycles) could improve the mixing quality equally without needing to extend the mixing cycles due to more emerging turbulences. For other inks, cell types or cell numbers, minor adjustments may be necessary to ensure optimal cell viability. To ensure comparability of both inks in this study, the mixing protocol designed for plasma-alg-mc was applied again. The viscosity and shape fidelity ratios were inside the defined optimal range, showing only small divergences from the optimal condition. These findings indicate the possible application of the mixing unit for various bioinks with only little changes to the proposed protocol. It was also demonstrated in fluorescence experiments that the mixing insert is necessary to obtain a high mixing quality (Figure S2). Remarkably, the cell viability after mixing and plotting in the gellan-based ink is not significantly lower than in the plasma-based one, even though the material became increasingly viscous over the course of the mixing, indicating a higher shear stress during the mixing. This further supports the hypothesis that the duration of shear stress might have a greater effect on cells than its strength.

Diamantides et al. and Gillispie et al. both demonstrated no significant influence of cell densities below 100 × 10^6^ cells/mL on the viscosity of low viscosity bioinks [29,30]. Based on these findings, we assumed that the presence of cells has no influence on the mixing behaviour using the mixing unit, which is evidenced by identical plotting properties of inks regardless of the presence of cells. Here, we used approximately 2 × 10^6^ cells per gram material, which is the established number used for MSC in our lab. Additionally, we could show a homogenous distribution for cell numbers up to 30 × 10^6^ (data not shown). It can thus be reasoned that for most cell concentrations relevant for biofabrication purposes, the mixing unit can provide good mixing results.

Next to the application for mixing already established highly viscous bioinks, providing reproducible and homogenous results, there are various applications for the developed static mixer unit. Although, in this paper, only the survival of one cell type during the mixing process was investigated, we experienced in several lab projects that various mammalian cell types but also yeasts, bacteria or cells from the plant kingdom show a similarly favourable response. Additionally, we predict that this mixing device is suitable for particle-containing bioinks, for example bioinks with bioglasses, calcium phosphates, or magnetic particles [40,41,42]. Future research should consider the static mixing unit for the homogenous mixing of such composite bioinks. Finally, this closed device could be useful for bioink preparation under GMP or microgravity conditions in space when the utilization of open containers for mixing is not applicable [25]. We invite other groups to share their experience applying the design with us using the provided STL file for further optimisation.

## 4. Conclusions

In conclusion, a novel and reproducible method for the homogenous mixing of highly viscous biomaterial inks and cell suspension was developed. In handling and functionality, it is comparable to commercially available static mixing units for low viscosity bioinks while also allowing modifications of the mixing unit to suit individual needs. A clear limitation of this approach is that the mixing device presented is still a manual method with user-dependent mixing speed. Nevertheless, the presented approach does provide one standardized step in the process of preparing a bioink. Using the mixing unit, the achieved cell viabilities were higher than those achieved by the established method using a spatula. Remarkably, these results were found for two very different high viscosity bioinks, indicating an application for a wide range of suitable bioinks. It was found that in order to qualify the mixing success, the combination of the two different parameters viscosity and shape fidelity was necessary to guarantee reliable results. These findings could be further confirmed by the analysis of the visual homogeneity.

## 5. Materials and Methods

### 5.1. Materials

Fresh frozen human plasma was provided by a local blood bank (German Red Cross–Blood Donation Service North-East, Dresden, Germany). The plasma of 5 donors was pooled for the experiments to even out differences in coagulation factor concentrations between donors. Alginic acid sodium salt from brown algae (M/G ratio 1:2) and methylcellulose (M0512, molecular weight ≈ 88 kDa, 4.000 cP·s) were purchased by Sigma-Aldrich (Frankfurt, Germany). Alginate (alg) and methylcellulose (mc) powders were sterilized by steam autoclaving at 121 °C for 20 min before usage. Strontium chloride (SrCl_2_) was obtained from Roth (Frankfurt, Germany). The used gellan gum was obtained under the trade name gelzan from Sigma-Aldrich (Germany) and the cell culture medium (Mesenchymal Stem Cell Medium Kit enhanced xeno-free, MSCGMx) used for crosslinking (“crosslinking medium”) was obtained by Pelo Biotech (Planegg, Germany). As cell culture medium for the plasma-alg-mc experiments, Dulbecco’s modified eagle’s medium (DMEM, Life technologies) (“cell culture medium”), containing 10% fetal calf serum (FCS, Corning, NY, USA), 100 U·mL^−1^ penicillin and 100 μg·mL^−1^ streptomycin (Life Technologies Corporation, MA, USA) was used. High temp V2 resin was obtained from Formlabs (Somerville, MA, USA). Green fluorescent protein (GFP) solution in a concentration of 300 µg/µL was obtained from EMD Millipore (EMD Millipore, CA, USA).

### 5.2. Static Mixer Production

All mixers were designed using computer-aided design (CAD) software and printed using Formlabs’ high temp V2 resin on a Formlabs Form 3 SLA printer with a layer height of 50 µm. Post processing included washing the parts in 2-propanol (Roth, Frankfurt, Germany) and curing according to manufacturer’s protocols. The STL file of the optimal static mixer design Screw-like v2 (“HighVisc”) is provided in the Supplementary Materials.

### 5.3. Ink Preparation

The plasma–alginate–methylcellulose (plasma-alg-mc) ink was prepared as described previously [10]. The process can be summarised briefly, as follows: alg was dissolved at a concentration of 30 mg × mL^−1^ directly in thawed human plasma. Afterward, mc was added at a concentration of 90 mg × mL^−1^. The blend was homogenized by gentle manual stirring using a spatula and was immediately processed further. For the gellan gum ink, 3 g of gellan gum was dissolved in 100 mL deionized water and kept overnight at 90 °C to ensure complete solution. Before using it in experiments, the solution was cooled down to room temperature to ensure comparability.

### 5.4. The Mixing of Cell Culture Medium and Biomaterial Ink

For experiments using plasma-alg-mc, 3 g of plasma-alg-mc were carefully transferred into a 5 mL single-use syringe with Luer lock system (B. Braun Melsungen AG, Melsungen, Germany). A total of 100 µL cell culture medium (DMEM) per gram material was transferred into a second syringe. Both syringes were connected using one of the static mixers and then both liquids were carefully mixed by moving the contents of the syringes back and forth, each passing of the material through the mixing unit representing one cycle. A set number of mixing cycles was performed, each transfer was timed to take 20 s to maintain a uniform flow. Subsequently, the ink was either transferred into a 10 mL plotting cartridge (Nordson EFD, Oberhaching, Germany) by connecting it to the static mixer before the last mixing cycle or was used for rheological analysis. As control, the medium and biomaterial ink were mixed manually within a beaker using a spatula as described before [10]. The gellan gum-based biomaterial ink was produced in the same way, substituting the standard cell culture medium with crosslinking medium MSCGMx.

### 5.5. Homogeneity of the Model Bioinks

#### 5.5.1. Rheology

Several cell-free bioink variants consisting of plasma-alg-mc and cell culture medium DMEM or gellan gum ink and crosslinking medium MSCGMx were evaluated for their rheological behaviour (Rheotest RN 4, Medingen, Germany). The viscosity was determined by a rotational measurement using a plate-plate geometry (d = 50 mm) with a gap distance of 0.1 mm at 25 °C. A constant shear rate of 10 s^−1^ was fed for 60 s. To evaluate the homogeneity of a given mixture of hydrogel and medium, two samples of each ink were rheologically analysed directly after mixing. Approximately 1 mL of ink from the tip of the syringe was measured, then 1 mL of ink was discarded, and the final 1 mL, from the bottom of the syringe, was analysed again. The ink’s homogeneity was determined by averaging the viscosity of both samples and then calculating the ratio of η_End_/η_Start_. The standard deviation was then determined by calculating the Gaussian error propagation of both averages.

To visualize the homogeneity after various mixing cycles, 30 µL green fluorescent protein (GFP) solution with a stock concentration of 300 µg/µL were added to 270 µL of cell culture medium or crosslinking medium. Then, the biomaterial inks and their respective GFP-enriched media were mixed using different mixing units as described above while taking fluorescence images (Keyence BZ-X800 fluorescent light microscope, Keyence, Ōsaka, Japan) of the syringes after every mixing cycle. Since the mixing apparatus was too big to be measured in one picture, the stitching function of the Keyence software was used to combine several taken images into one. Using the imaging software Fiji [43], the intensity of fluorescence over the entire length of the filled syringe was measured using the software’s “Plot Profile” method. The obtained values were then normalized to the respective intensity maximum to compensate for differences in measured intensity.

#### 5.5.2. Scaffold Fabrication

The model bioink consisting of plasma-alg-mc and cell culture medium was transferred into a 10 mL plotting cartridge (Nordson EFD, Oberhaching, Germany) either using the static mixer (experiment) or a spatula (control) and plotted by a pneumatic-driven extrusion plotter (Bioscaffolder 3.1, GeSiM mbH, Radeburg, Germany). The inks were extruded through dosing needles (d = 410 µm; Globaco, Rödermark, Germany) with 40–45 kPa air pressure, a plotting speed of 12 mm s^−1^ and a layer height of 0.26 µm. Cuboidal scaffolds (base area 16 × 16 mm^2^) with a strand distance of 3 mm, four layers and a layer-to-layer orientation of 90° were fabricated. For the gellan gum-based bioink, ink and crosslinking medium were mixed using the “Tube” and “Screw-like v2” static mixer and then processed as described above. For the determination of shape fidelity, three scaffolds were plotted using ink directly from the tip of the cartridge (“Start”). Subsequently, ink was extruded for 30 s before three more scaffolds were plotted (“End”), all scaffolds, without crosslinking, were then imaged using a Leica M205 C stereo microscope equipped with a DFC295 camera (Leica Microsystems, Wetzlar, Germany). The strand width d was determined using the image processing software Fiji (Version 1.52p). For analysis, the ratio of d_End_/d_Start_ was calculated.

### 5.6. Bioprinting

#### 5.6.1. Cell Expansion

Immortalized human mesenchymal stem cells (MSC) expressing human telomerase (hTERT) [44] were expanded in monolayer culture at 37 °C and 5% CO_2_ in DMEM cell culture medium, the medium was changed twice a week.

#### 5.6.2. Bioprinting of Cell-Laden Constructs

Cells were harvested from cell culture flasks and afterwards centrifuged and resuspended in 100 µL cell culture medium per gram of hydrogel to a final cell concentration of 1.82 × 10^6^ cells × g^−1^ bioink. Subsequently, the cell suspension was mixed into the hydrogel, either by transferring it into a 5 mL syringe and using a static mixer as described in chapter 5.3 or manually using a spatula and mixing both components within a sterile 50 mL centrifugation tube (Cellstar^®^, Greiner-Bio-One GmbH, Frickenhausen, Germany). For each type of mixing unit, three scaffolds were printed using material from the start and the bottom of the cartridge, respectively. Up to this point, the procedure was the same for both bioinks. After plotting, the plasma-alg-mc scaffolds were crosslinked for 10 min in a bath of 70 mM SrCl_2_ solution in a sterile environment and subsequently transferred into cell culture medium. The scaffolds consisting of the gellan gum bioink were submerged and cultured in crosslinking medium after plotting.

#### 5.6.3. Assessment of the Cell Viability

Cell viability was investigated using live/dead staining with calcein-AM/ethidium homodimer-1 (LIVE/DEAD Viability/Cytotoxicity Kit for mammalian cells, Thermo Fisher Scientific, Waltham, MA, USA) following the manufacturer’s protocol. Confocal laser scanning microscopy (cLSM, Leica TCS SP5) and fluorescence microscopy (Keyence BZ-X800 fluorescent light microscope) were used to determine quantitative cell viability of MSC at day 0 (n ≥ 4) immediately after printing and crosslinking. Cell viability was assessed by semiautomatic area determination of living and dead cells with thresholds applied to all image stacks (n ≥ 12 per group) using ImageJ (Fiji, Version 1.52p). The viability was defined as the ratio of the area of living cells divided by the sum of areas of live and dead cells. Here, all six plotted constructs per mixing unit were analysed and the average cell numbers were used.

### 5.7. Statistical Analysis

All values were evaluated by one-way analysis of variance (ANOVA), followed by Tukey’s multiple comparison test with GraphPad Prism 8 software. Significant differences were assumed at *p* < 0.05.

## Data Availability

Not applicable.

## Supplementary Materials

The following are available online at https://www.mdpi.com/article/10.3390/gels7040227/s1, Figure S1: Cell viability (n ≥ 9, mean ± standard deviation, ** *p* < 0.05, ** *p* < 0.01, *** *p* < 0.001 for the mixing with several mixing units and spatula as a reference analysed directly after printing of plasma-alg-mc scaffolds, Figure S2: Mixing of gellan-based biomaterial ink with green fluorescent protein dissolved in cell culture medium using the “Tube” or “Screw-like v2” mixer unit; (a): fluorescence microscopic images of both syringes before mixing (c = 0) and after several mixing steps (c = 19). (b): normalized intensity graphs measured over the entire cartridge length. Scale bar: 15 mm.
