# Supplementary figures and images for: Homogeneous and Reproducible Mixing of Highly Viscous Biomaterial Inks and Cell Suspensions to Create Bioinks
Source: Gels. 2021 Nov 23;7(4):227. doi: 10.3390/gels7040227 (PMC8628813; doi:10.3390/gels7040227)

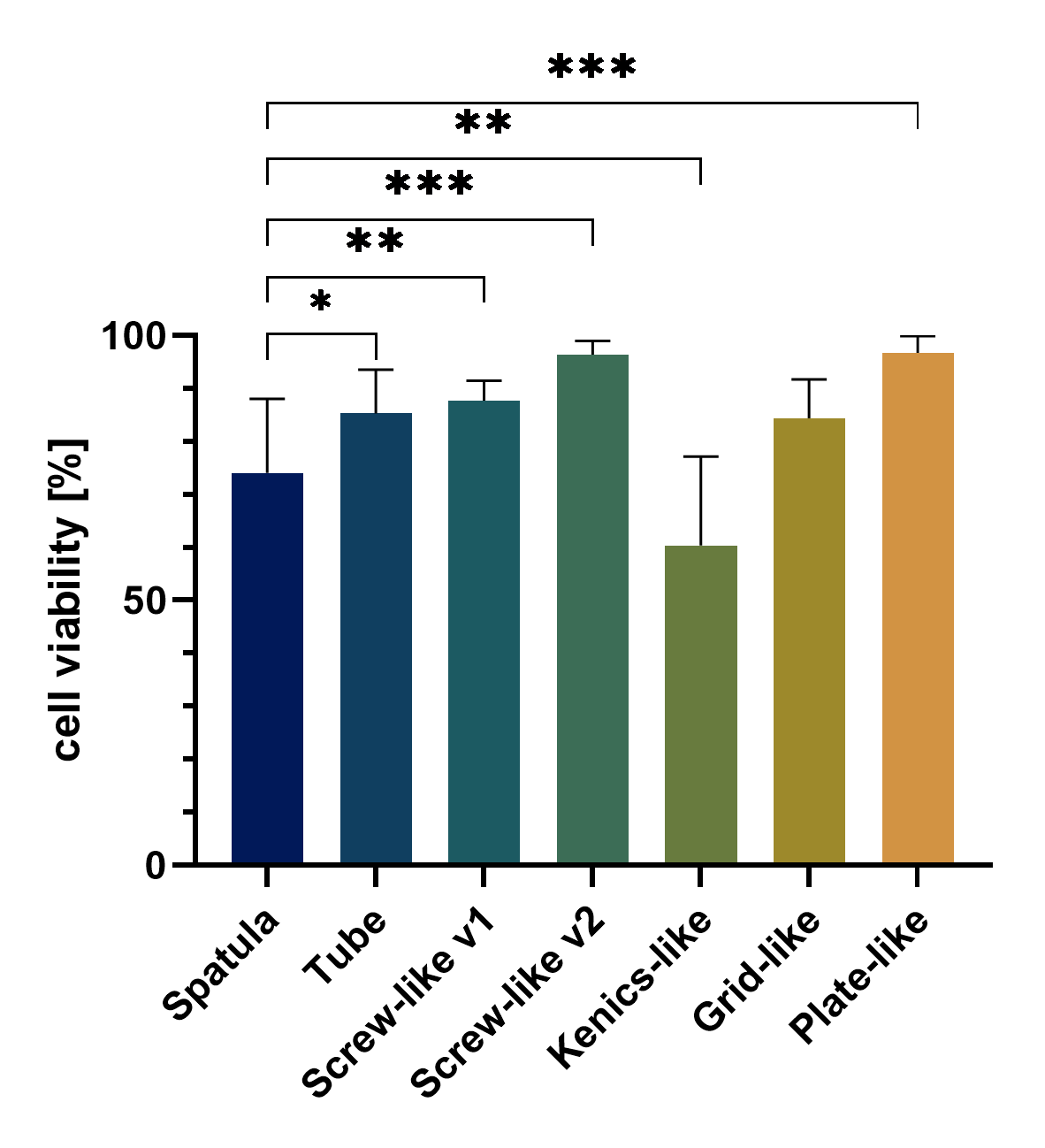

Supplement: Supplementary file 1 [file gels-07-00227-s001.zip › FigureS1.tif]

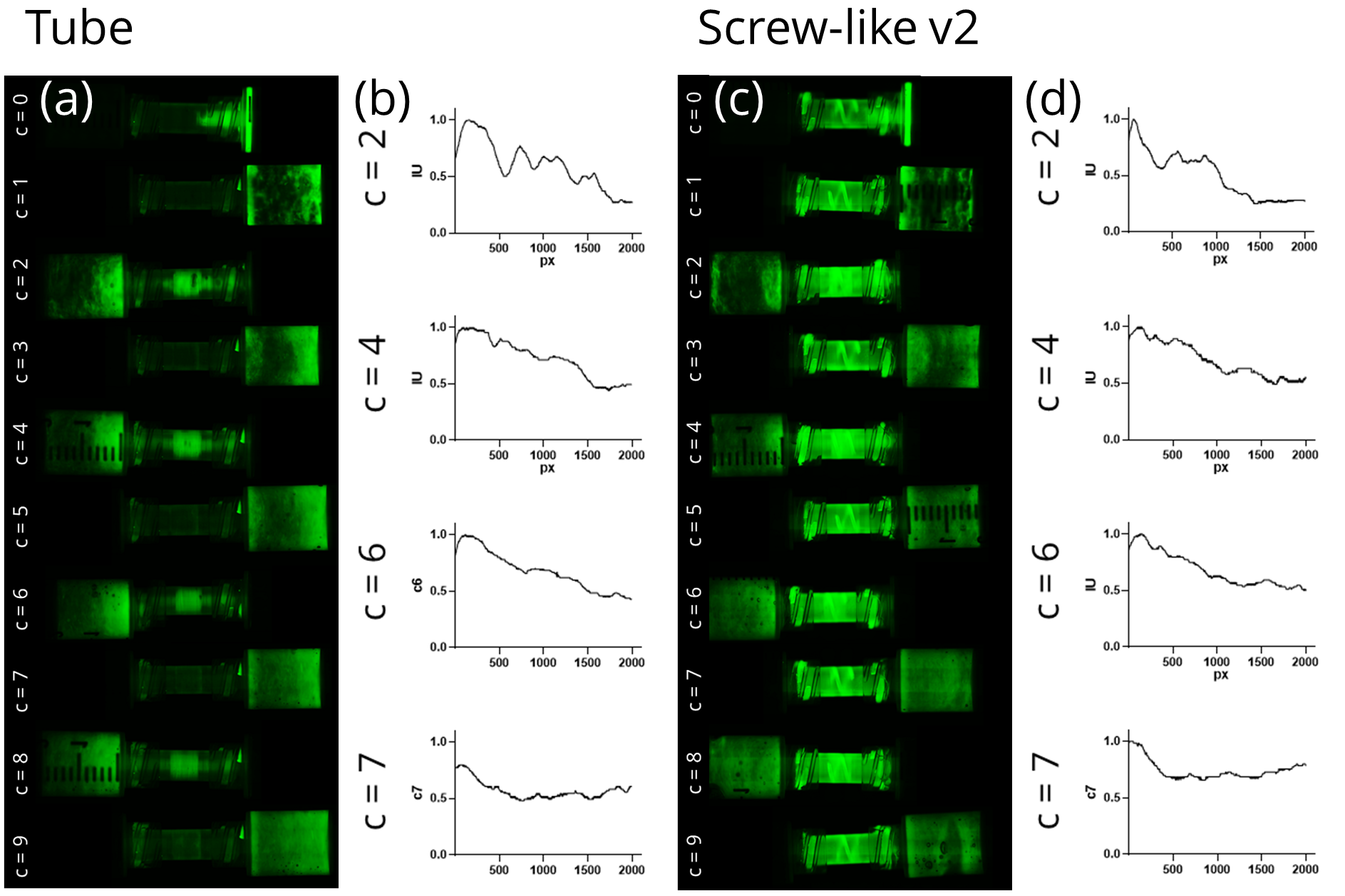

Supplement: Supplementary file 1 [file gels-07-00227-s001.zip › FigureS2.tif]
